# A phase I/II escalation trial design T-RAD: Treatment of metastatic lung cancer with mRNA-engineered T cells expressing a T cell receptor targeting human telomerase reverse transcriptase (hTERT)

**DOI:** 10.3389/fonc.2022.1031232

**Published:** 2022-11-10

**Authors:** Sólrún Melkorka Maggadóttir, Gunnar Kvalheim, Patrik Wernhoff, Stein Sæbøe-Larssen, Mona-Elisabeth Revheim, Dag Josefsen, Sébastien Wälchli, Åslaug Helland, Else Marit Inderberg

**Affiliations:** ^1^ Translational Research Unit, Department of Oncology, Section for Cellular Therapy, Oslo University Hospital, Oslo, Norway; ^2^ Department of Medical Genetics, Institute of Clinical Medicine, University of Oslo and Oslo University Hospital, Oslo, Norway; ^3^ Division of Radiology and Nuclear Medicine, Oslo University Hospital, Oslo, Norway; ^4^ Department of Clinical Medicine, University of Oslo, Oslo, Norway; ^5^ Department of Cancer Genetics, Institute for Cancer Research, Oslo University Hospital, Oslo, Norway; ^6^ Department of Oncology, Oslo University Hospital, Oslo, Norway

**Keywords:** TCR immunotherapy, hTERT, NSCLC, mRNA electroporation, solid tumor immunotherapy, ACT

## Abstract

**Background:**

Adoptive cellular therapy (ACT) with genetically modified T cells aims to redirect T cells against resistant cancers through introduction of a T cell receptor (TCR). The Radium-4 TCR was isolated from a responding patient in a cancer vaccination study and recognizes the enzymatic component of human Telomerase Reverse Transcriptase (hTERT) presented on MHC class II (HLA-DP04). hTERT is a constitutively overexpressed tumor-associated antigen present in most human cancers, including non-small-cell lung cancer (NSCLC), which is the second most common type of cancer worldwide. Treatment alternatives for relapsing NSCLC are limited and survival is poor. To improve patient outcome we designed a TCR-based ACT study targeting hTERT.

**Methods:**

T-RAD is a phase I/II study to evaluate the safety and efficacy of Radium-4 mRNA electroporated autologous T cells in the treatment of metastatic NSCLC with no other treatment option. Transient TCR expression is applied for safety considerations. Participants receive two intravenous injections with escalating doses of redirected T cells weekly for 6 consecutive weeks. Primary objectives are safety and tolerability. Secondary objectives include progression-free survival, time to progression, overall survival, patient reported outcomes and overall radiological response.

**Discussion:**

Treatment for metastatic NSCLC is scarce and new personalized treatment options are in high demand. hTERT is a tumor target applicable to numerous cancer types. This proof-of-concept study will explore for the first time the safety and efficacy of TCR mRNA electroporated autologous T cells targeting hTERT. The T-RAD study will thus evaluate an attractive candidate for future immunotherapy of solid tumors.

## Introduction

T cells are effector cells of the adaptive immune system that can recognize and kill malignant cells. In addition, they also play the role of master regulator of the immune response and interact with other cells to induce effective antitumor responses.

Adoptive cell Therapy (ACT) using genetically modified T cells relies on the use of T cells to destroy malignant tumor cells. This can be achieved by introducing a receptor, either T cell receptor (TCR) or Chimeric Antigen Receptor (CAR) to enable specific recognition of a target antigen on the surface of cancer cells. Target antigen recognition is complex with clustering of the receptor leading to T cell activation. Since CARs are mainly antibody-based molecules that depend on direct contact with the surface antigen, their targets are restricted to plasma membrane proteins. On the other hand, TCRs recognize peptides presented by the Major Histocompatibility Complex (MHC or Human Leukocyte Antigen (HLA)) and since all proteins in a cell are degraded and presented on MHC, independently of their location, TCRs can potentially detect peptides generated from any protein. A few CARs have been approved for clinical use with impressive results, mainly in hematological cancers, to date no TCRs have been clinically approved. This is largely due to their MHC restriction which prevents their universal use. However, some recent studies have demonstrated clinical responses from the application of TCR modified T cells in solid tumors (1–3). One recent case report demonstrated significant results in pancreatic cancer targeting the KRAS G12D mutation (4), although overall ACT treatment of solid tumors has had limited success to date. One solution would be to isolate collection of TCRs with specificity for MHC alleles that are frequently expressed in human populations, such as HLA-DP4 (5).

hTERT (human Telomerase Reverse Transcriptase) is a tumor-associated antigen (TAA) that is constitutively overexpressed in >90% of human cancer cells (6–9), including most solid cancers. hTERT overexpression is important for metastasis and epithelial-mesenchymal transition (EMT) in cancer cells (10) as well as for cancer stem cells that likely impact risk of cancer relapse and treatment resistance (11, 12). Taken together the above makes hTERT an attractive target for T cell immunotherapy.

**Figure 1:**
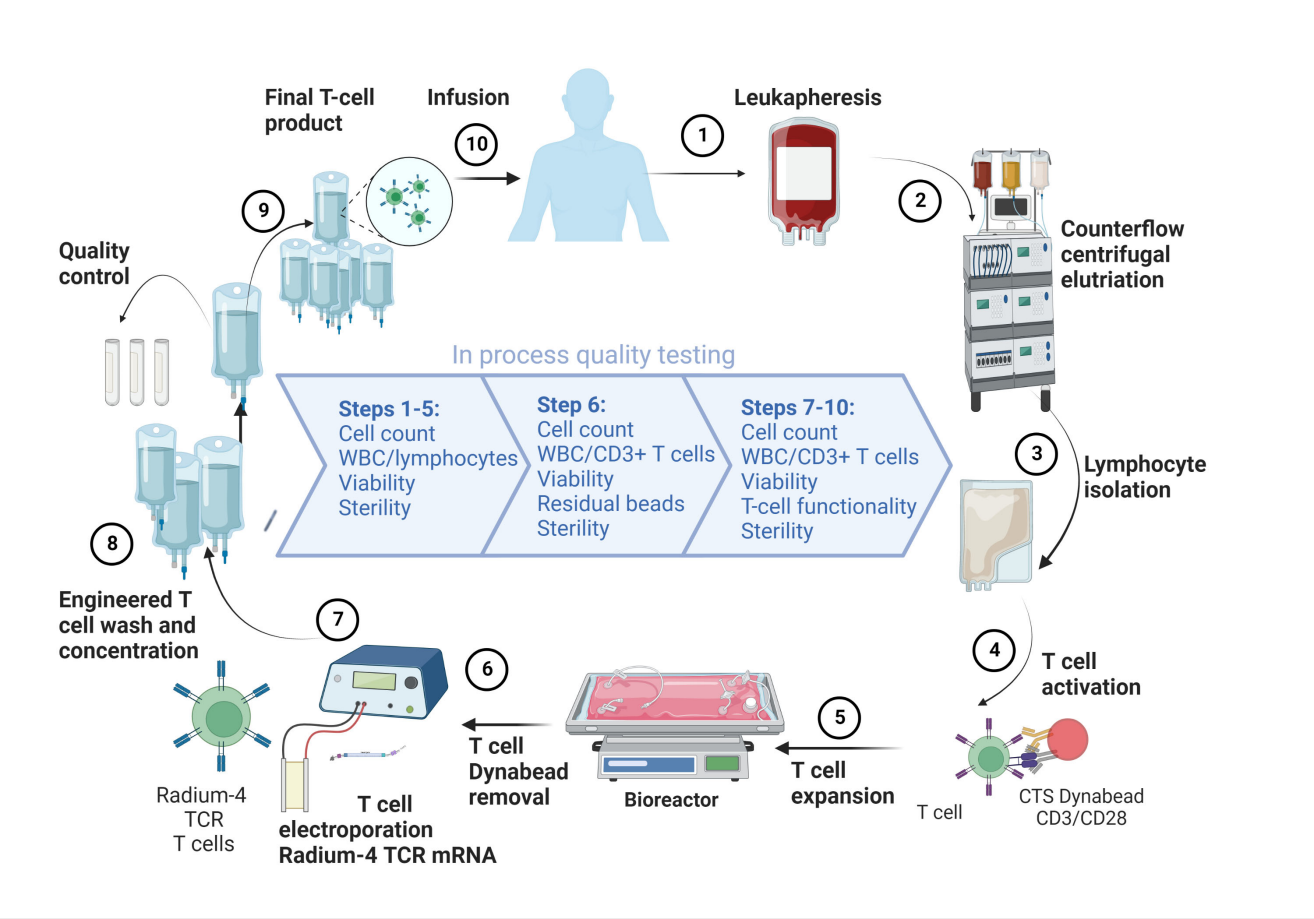
Overview of the GMP-production platform for TCR T cell therapy. Created using BioRender.com.

Selection and modification of TCRs for ACT use is a very important and delicate process given the demonstrated off-target activity by enhanced TCRs resulting in fatal toxicities in prior studies (13, 14). Our approach is to use tumor-specific TCRs identified and isolated from T cell populations in responding patients from cancer vaccination trials (15–18). T cells are then isolated from the patient, expanded *in vitro* and electroporated with mRNA encoding the tumor-specific TCR, see **Figure 1**. Since such TCRs have already passed thymic selection and are unmodified they are not expected to cause off-target toxicities in MHC matched individuals and should have an excellent safety profile as suggested by our recent protocol using a TCR targeting a neoantigen expressed in a subgroup of colon cancer tumors and presented by the common HLA-A2 allele (ClinicalTrials.gov: NCT03431311, manuscript in preparation) (19). We have previously reported on the isolation, pre-clinical efficacy testing and safety validation of our MHC class II restricted TCR, Radium-4, that recognizes the enzymatic component of hTERT (20). The Radium-4 TCR is HLA-DP04 and given that this MHC II alleles are expressed in about 76% of the Caucasian population the Radium-4 TCR is widely applicable (5, 21, 22)

**Figure 2:**
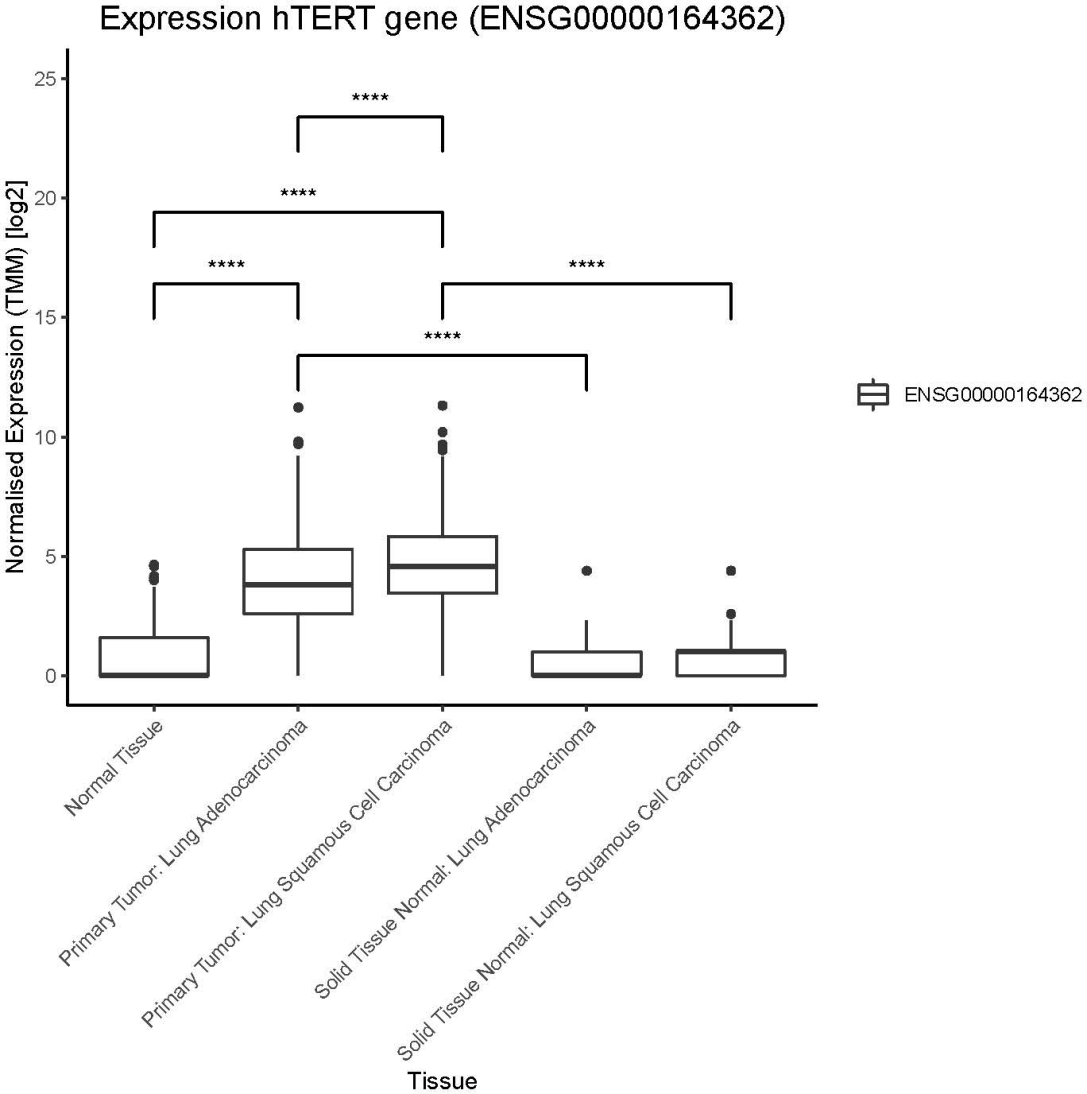
hTERT expression in lung cancer patients. Expression of hTERT in normal tissue (GTEX), primary tumors and solid tissue normal (TCGA). Solid tissue normal represents normal tissue from the same cancer patients. Significant differences between normal tissue and primary tumors, as well for solid tissue normal (not indicated in figure). Number of samples per group: normal tissue: 288, primary tumor lung adenocarcinoma: 513, primary tumor lung squamous cell carcinoma: 498, solid tissue normal lung adenocarcinoma: 59, solid tissue normal lung squamous cell carcinoma: 50. Significance tested using pairwise.wilcox.test, padj < 0.0001 indicated with (****). P value adjustment method: bonferroni.

In 2020 lung cancer was, world-wide, the most common cause of cancer death and the second most common type of cancer diagnosed (23). The majority of lung cancer (75-80%) is non-small cell lung cancer (NSCLC) and to date the only treatment with a significant chance of long-term survival is surgery and stereotactic radiotherapy. Unfortunately, most lung cancers are diagnosed at an advanced stage with only a third of patients being treated with curative intent. Palliative treatment options include however, surgery, radiotherapy, checkpoint inhibitor- and chemotherapy depending on clinical symptoms and extent of disease. Overall survival in advanced NSCLC is quite poor, with the 5-year survival in patients with metastatic disease being <10% (24). Immune checkpoint inhibitors have improved survival in NSCLC, demonstrating the importance of uninhibited immune responses in this disease, but sadly only 20-30% of advanced NSCLC patients respond to that treatment and resources for relapsing patients are scarce and new options are much needed (25–27) The dominant immunogenic antigens in NSCLC remain largely unknown and to date no ACT using an MHC class II restricted TCR has been published. A single trial of ACT using CARs in NSCLC was recently published targeting the epidermal growth factor receptor (EGFR) using a non-viral transposon system (28). Three publications using CARs for thoracic malignancy (mesothelioma) have been published. Two used local delivery of retrovirally modified CAR T cells (29, 30) and only one used mRNA electroporation (31). In all of these studies safety and tolerability was demonstrated, while efficacy was variable. Nevertheless, hTERT is a valid target in NSCLC where the same hTERT epitope has previously been targeted by vaccination (15, 32). Our analysis of hTERT expression by RNAseq analysis demonstrated higher expression in primary lung tumor tissue than that seen in normal solid lung tissue from the same patients, see **Figure 2**. This was seen for both squamous cell carcinoma and adenocarcinoma, the two most common subtypes of NSCLC, although the normalized expression was significantly higher in squamous cell carcinoma. Furthermore, it is noteworthy that hTERT expression showed a trend toward higher expression in adjacent normal tissue in squamous cell carcinoma as compared to normal tissue. This was not seen for adjacent normal tissue in adenocarcinoma, see **Figure 2**. In summary, advanced NSCLC is an immunogenic tumor where new treatments are in high demand and hTERT is an attractive target for immunotherapy in NSCLC given its elevated expression in NSCLC tumor tissue.

To explore new treatment options for metastatic NSCLC we designed the T-RAD study where we adoptively transfer the hTERT specific TCR, Radium-4, to autologous T cells of participants by mRNA-electroporation. This allows for transient expression of the Radium-4 enhancing the safety of this first-in-human phase I/II study. Participants will receive two intravenous injections weekly of redirected T cells in escalating doses, similar to that applied in previous trials for chimeric antigen receptor (CAR)-based therapy (31). The treatment is given for 6 consecutive weeks. This is the first clinical study using an hTERT specific TCR for immunotherapy and the primary objectives of the T-RAD study are safety and tolerability.

In summary, we describe a phase I/II escalation trial design, T-RAD, as a first-in-human treatment of NSCLC using adoptive transfer, of the Radium-4 TCR, by mRNA-electroporation to autologous T-cells.

## Methods

### hTERT expression analysis

The RNA expression of hTERT was analyzed in the TCGA research network (33) using samples from cohorts for lung cancer (primary tumors) and normal solid tissue (normal sample from the same cancer patient). The normal tissue samples were taken from The Genotype-Tissue Expression (GTEx) project (34). In this study RSEM expected count data from TCGA and GTEx was downloaded, cohort TCGA TARGET GTEx, from University of California, Santa Cruz (35, 36). For “sample-to-sample” comparisons of gene expression, expected counts from RSEM were imported into edgeR package (R-version R4.0.2) (37). Counts were ‘delogged’ and normalized by using the Trimmed Mean of M values (TMM) method in edgeR. The TMM values were log2 transformed for presentations and comparisons. The pairwise.wilcox.test was used to test statistical significance between the sample groups, a padj value < 0.05 was considered significant. The Bonferroni method was used for p value adjustment.

### Study design and treatment schedule

**Figure 3:**
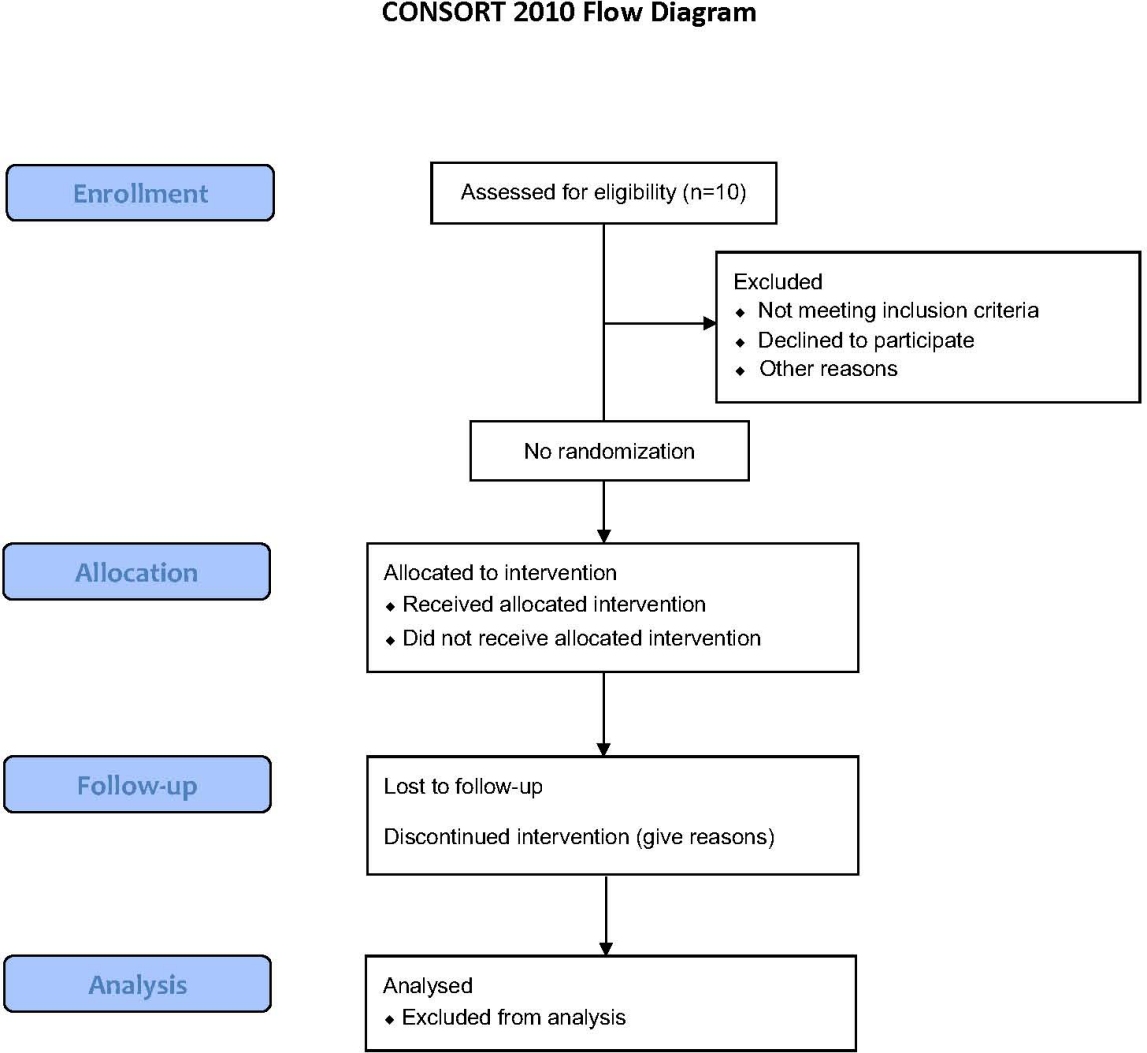
Flow Diagram for the T-RAD study created using the CONSORT 2010 Flow Diagram.

T-RAD is an exploratory, single arm, open-label, non-randomized phase I/II clinical study evaluating the safety, tolerability, and feasibility of administering autologous T cells transiently expressing the HLA-DP04 (MHC class II) restricted Radium-4 TCR targeting hTERT for the treatment of metastatic NSCLC, see **Figure 3**. Transient expression of Radium-4 will be achieved by mRNA electroporation of the autologous T cells as previously described (20, 38).

**Figure 4:**
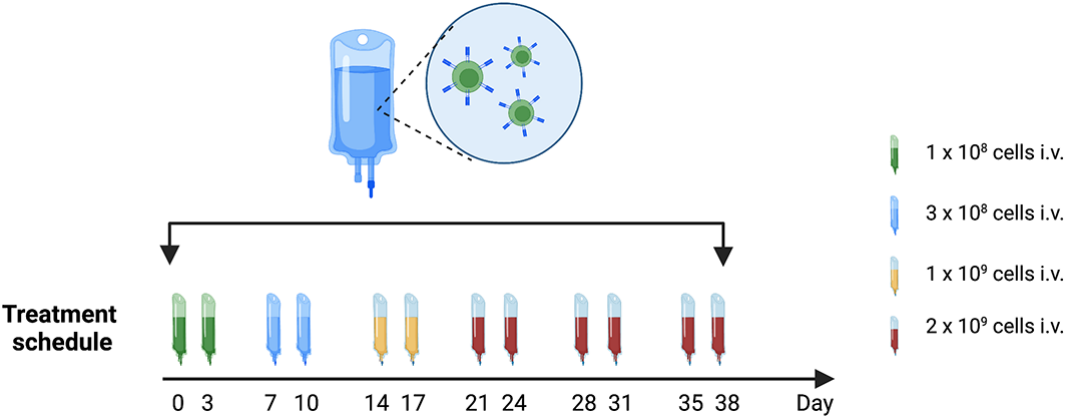
Treatment schedule with timing and doses of Radium-4 T cell infusions in the T-RAD study. Created using BioRender.com.

Patients will receive treatment according to our treatment protocol that includes 12 intravenous infusions, evenly distributed over the course of 6 weeks. Doses are escalating starting at 1 x 10^8^ cells and reaching 2 x 10^9^ cells, see **Figure 4**. Our goal is to treat 10 patients with Radium-4 redirected T cells.

### Objectives and rationale

The primary objective is to evaluate toxicity, safety, and tolerability of the treatment. This will be achieved through monitoring and documentation of incidence, nature and severity of adverse events graded according to National Cancer Institute Common Terminology Criteria for Adverse Events (NCI CTCAE) v4.0. In addition, clinical assessment, including vital signs and Eastern Cooperative Oncology Group (ECOG) performance status, hematology and clinical chemistry and plasma cytokine laboratory results will be monitored.

Secondary objectives are to assess clinical response to the treatment through progression-free survival (PFS) and radiological overall response rate (ORR) as defined by RECIST 1.1/iRECIST for CT scan results and EORTC/PERCIST for FDG PET scan results, see further in section on data gathering and analysis. Additionally, time to progression (TTP) and overall survival (OS) will be evaluated. Finally, patient reported outcome measurements (PROMs) in terms of cancer symptoms and health-related quality of life will be applied in the form of EORTC questionnaires (European Organization for Research and Treatment of Cancer).

Exploratory objectives are assessment of changes in T cell infiltration into tumor tissue, spatial orientation of such changes as well as PD-L1 and PD-1 expression in biopsies pre-treatment and upon progression. See **Table 1** for list of objectives.

**Table 1:** Primary, secondary, and explorative objectives in the T-RAD study.

**Primary objectives:**
Assessment of toxicity and tolerability Assessment of safety
**Secondary objectives:**
Assessment of clinical response to T-RAD treatment: progression-free survival (PFS), radiological overall response rate (ORR), time to progression (TTP) and overall survival (OS) Patient reported outcome measures (PROMs) and health related quality of life (QoL)
**Exploratory objectives:**
Changes in the immunological milieu in the tumor tissue: T cell infiltration in tumor tissue, spatial orientation of immune cell infiltration and PD-L1 and PD-1 expression in tumor tissue

Due to the wide applicability of hTERT as a TAA in many solid cancers such as NSCLC, and the commonality of its associated MHC alleles, the Radium-4 is expected to have effective anti-tumor effects. Its origin from a patient in a cancer vaccine trial enhances the safety profile of this TCR and our approach of mRNA-electroporation further supports the tolerability of the treatment in T-RAD.

Introducing a tumor-antigen specific TCR into an autologous T cell by means of mRNA electroporation allows for expression of a functional TCR that recognizes an overexpressed TAA, such as hTERT. The benefit of using mRNA to introduce such TCRs into T cells is that their expression is transient for a few days, allowing for quick cessation of effects should any cross-reactivity occur. Our prior preclinical evaluation demonstrated the ability of the Radium-4 TCR to recognize hTERT and to introduce efficient cellular killing and cytokine secretion both *in vitro* and in a xenograft mouse model where we demonstrated improved survival without toxicity (20).

### T cell manufacture

**Figure 5:**
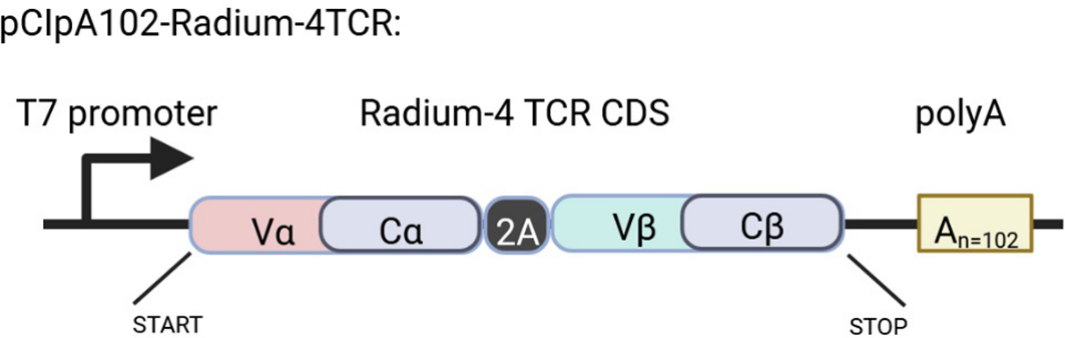
Schematic representation of the Rad4 TCR construct in the pCIpA102 expression vector used for the *in vitro* transcription of Radium-4 TCR mRNA. Created using BioRender.com.

For T cell manufacturing (see **Figure 1**) we will collect peripheral blood mononuclear cells (PMBCs) from participants by leukapheresis. At the GMP facility of the Department of Cellular Therapy at The Oslo University Hospital, Norway, lymphocytes will be enriched through elutriation (ELUTRA, TerumoBCT) and expanded using CD3/CD28 CTS™ Dynabeads™ (Thermo Fisher Scientific). Lymphocytes will be expanded in CellGro GMP DC medium (CellGenix, GmbH, Germany) supplemented with 5% human serum (PAN-Biotech, GmbH, Germany), Mucomyst (Mylan Healthcare Norway AS), gentamycin (Sanofi Aventis, France) and 100 U/mL interleukin-2 (IL-2) (Proleukin, Clinigen Healthcare Ltd, UK) for 10 days. Expanded T cells will be electroporated and transfected with *in vitro* transcribed (ivt) codon-optimized mRNA (clinical grade, produced at Oslo University Hospital, Norway) encoding the Radium-4 TCR, as previously described (20) and **Figure 5**. Following recovery in medium containing human serum the desired amount of T cells used for injection will be transferred to individual freezing bags, frozen and stored in DMSO-containing cryopreservation media in liquid nitrogen, vapor phase. At the time of infusion, frozen T cells will be thawed at the GMP facility and thereafter immediately transferred to the clinical ward and infused through an intravenous access. Quality-control test criteria will have to be met prior to infusion of the product pertaining to T cell count, viability, and absence of microbiological contamination. See **Table 2** for release criteria for the final cellular product.

**Table 2:** List of release criteria for each cellular product batch to be used in the T-RAD clinical study.

Parameter	Timepoint of testing	Specific criteria
**Microbiological contamination**	After T cell expansion, prior to and after electroporation, and the final frozen T cell product.	No contamination
**Viability**	Testing of final formulation, after electroporation	> 60%
**% CD3 cells**	After recovery, post electroporation	> 60%

### T cell potency testing

Surface detection of the Radium-4 TCR by flow cytometry is not possible since no commercial antibodies recognizing the TCR chains are available. Also, production of any stable peptide-MHC multimers has not been possible, which is typical for MHC II restricted TCRs. In our prior clinical protocol (ClinicalTrials.gov: NCT03431311) where we used an MHC class I restricted TCR we were able to confirm good and consistent electroporation results. This was done by detection of surface expression of the TCR by antibody staining. In T-RAD cytokine production by flow cytometry and/or bioluminescence (BLI-) based cytotoxicity assay *in vitro* will be performed on expanded and electroporated T cells. This will be done prior to patient infusion and a minimum threshold of 60% specific killing by 10 hours used to ensure a functional product.

**Figure 6:**
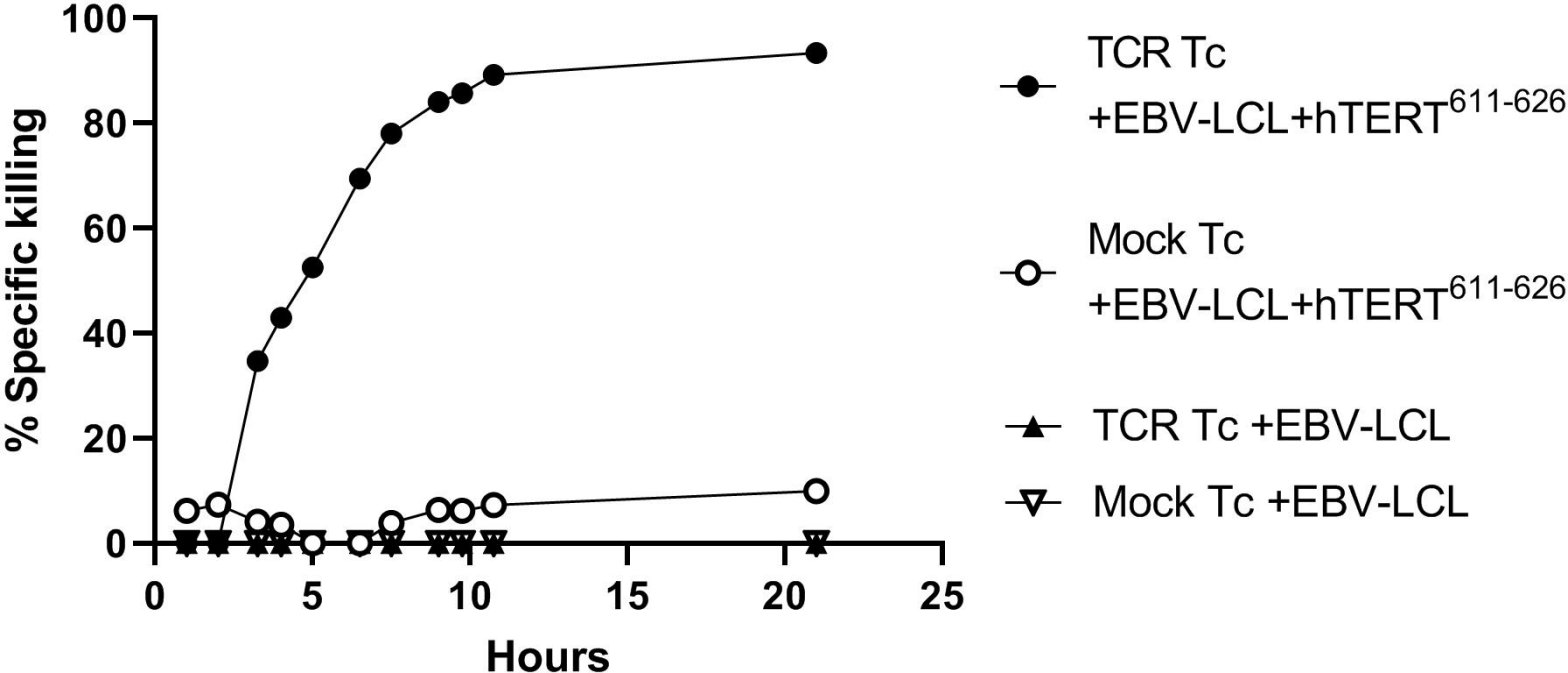
TCR induced killing of hTERT-peptide presenting target cells. TCR-transfected and mock-transfected T cells were tested using a luciferase expressing HLA-matched Epstein Barr virus (EBV) transformed lymphoblastoid cell line (EBV-LCL). The cells were loaded with the corresponding peptide hTERT^611-626^. Effector:target (E:T) ratio was 30:1 and luminescence was measured with a luminometer at defined time points. All conditions were tested in triplicates and after 2 months of therapeutic cell storage. This will be done prior to patient infusion in the T-RAD study and a threshold of ≥ 60% specific killing at 10hrs used for a functional product.

In **Figure 6** the BLI-based cytotoxicity assay was performed as previously described (18). Briefly, the functionality of both hTERT TCR-transfected and mock-transfected T cells was tested. For this a luciferase expressing Epstein Barr virus-transformed lymphoblastoid cell line (EBV-LCL) was used. The EBV-LCL cell line was HLA-matched to allow presentation and recognition of the hTERT peptide. The EBV-LCL cells were loaded with the cognate peptide hTERT^611-626^. As expected, mock transfected T cells did not demonstrate significant target killing. Specific killing by TCR-transfected cells could be observed after 3-4 hours of co-culture and was saturated after 10-12 hours, see **Figure 6**.

### Participant selection

We intend to treat and fully evaluate 10 patients with metastatic NSCLC > 18 years old of any race or gender with an ECOG score of 0-1 and > 3 months life expectancy. This phase I study will include and treat patients at Oslo University Hospital. HLA-typing will be performed by the National Tissue typing Laboratory. See **Table 3** for selected inclusion and exclusion criteria for the T-RAD clinical study.

**Table 3:** List of selected inclusion and exclusion criteria to be used in the T-RAD clinical study.

Inclusion criteria	Exclusion criteria
Metastatic NSCLC and an HLA-DP*04:01 or HLA-DP*04:02 genotype	Other metastatic malignancies
No other treatment option available	Other anti-tumor treatment < 4 weeks of first T cell infusion in T-RAD
Measurable metastatic disease according to RECIST 1.1/iRECIST	Steroid treatment, except substitution doses
ECOG performance score of 0-1	Significant cardiac or other medical illness that would severely limit activity or survival such as severe congestive heart failure, unstable angina or serious cardiac arrhythmia
Life expectancy of at least 3 months	Active infection requiring antibiotic therapy
Willing to provide blood samples and tissue biopsies for research	Known hypersensitivity to any of the components of the infusion product
Adequate organ function as defined in the treatment protocol	Patients who test positive for hepatitis B, C, HIV or syphilis
Women or men aged ≥ 18 years	Pregnant or breastfeeding
Signed informed consent	Any reason why, in the opinion of the investigator, that the patient should not participate in this clinical trial

### Safety measures and handling of potential toxicities

Administration of autologous Radium-4 T cell immunotherapy has a very good risk-benefit profile. No difference in the nature of the toxicity is to be expected from repeated doses vs a single dose although a stronger response could occur with more numerous doses. The dose regimen applied in the T-RAD trial is comparable to prior studies and the same we applied in our prior clinical study of Radium-1 TCR (ClinicalTrials.gov: NCT03431311, manuscript in preparation) with minimal toxicity (31). Patients enrolled in the T-RAD study will be treated in an inpatient ward and attended to by staff trained to monitor for and respond to emergencies, especially to the development of Cytokine Release Syndrome (CRS) or Tumor Lysis Syndrome (TLS). We have in place special protocols for treatment of both CRS and TLS.

All serious and non-serious adverse events will be monitored and graded according to NCI CTCAE v4.0. Clinical assessment (including vital signs, interim history, physical exam) and laboratory values will be monitored and evaluated prior to each infusion. All adverse events, during the study and for up to 30 days after the last dose, will be documented, reported, and recorded in the patient’s file. Study patients who have an adverse event will be followed until the event has resolved, is determined to be stable, patient withdraws consent or is lost to follow-up or if study treatment is determined to not be the cause of the adverse event. As an additional safety measure, the first two patients will be included with a minimum of 4 weeks between start of treatment. For treatment of adverse events the protocol refers to the Investigator’s Brochure (IB) for this study.

Although hTERT is transiently active in certain normal cell populations, such as stem cells, reproductive cells and intestinal epithelium, the enzyme is inactive in most normal cells in the adult (39–41). Importantly, normal tissues expressing hTERT lack MHC class II expression and should not be recognized or killed by T cells expressing the Radium-4 TCR and thus the risk of serious toxicities in from this is low. In our preclinical studies we did not detect any toxicity against bone marrow stem cells (20). Our data, and that from prior studies, indicate that the TCR expression following mRNA electroporation of T cells reaches its maximum within approximately 12-18 h. Thereafter, the TCR expression gradually decreases and reaches undetectable levels after approximately 4 days (20, 38). If the TCR T cells encounter their cognate peptide:MHC positive target cells *in vivo*, the TCR T cells will proliferate and thus lose expression of the introduced TCR even more rapidly. In our study T cell infusions will be administered twice weekly (every 3-4 days) and therefore Radium-4 TCR T cells will lose expression of the Radium-4 TCR prior the next infusion. Transient TCR expression following mRNA electroporation allows for rapid termination of T cell activation by cessation of T-cell infusions which is critical to quickly reduce the impact of any potential cross-reactivity or other toxicity. Additionally, by using mRNA electroporation no viral vectors are introduced, eliminating the risk of insertional mutagenesis.

Potential toxic effects to consider in T-RAD are twofold, firstly toxicity from cross-reactivity or off-tumor reactivity if the autologous Radium-4 TCR transfected T cells were to attack and kill autologous patient cells, and secondly cytokine release syndrome (CRS) and tumor lysis syndrome (TLS). The risk of these is expected to be very low, time to onset would be short and due to the transient nature of the TCR expression, reversibility of potential side effects is expected to be very fast. CRS results from activation and cytokine secretion by the infused redirected T cells, as well as from positive feedback loops leading to activation of other immune cells. CRS is a form of systemic inflammatory response syndrome (SIRS) and often occurs simultaneously with TLS which is caused by the rapid breakdown of dying tumor cells. As expected, risk of both CRS and TLS is the highest in the setting of high tumor burden and in hematological diseases. Due to the transient expression of Radium-4 on the autologous T cells in our study the expression will not be retained during proliferation in the participant and thus the occurrence of CRS is much less likely than seen with virally transduced T cells for immunotherapy (42, 43). In the single published study using mRNA electroporation CRS was not common (31) and in our own recent study using the Radium-1 TCR we did not observe any CRS symptoms (NCT03431311, manuscript in preparation). Given the above, should CRS or TLS symptoms occur the expectation is that these would quickly abort with termination of T cell infusions.

### Data gathering and analysis

The informed consent form for the T-RAD study includes blood and biopsy sampling, immunological characterization, gene profiling, radiological analysis, and studies of tumor immune cell infiltrate. Blood samples will be collected pre-, during and post-treatment and both PBMCs and plasma will be frozen down. Tumor biopsies will be collected pre- and post-completion of treatment. Patients will sign an informed consent that includes all the above sampling and analyses.

Data on adverse events according to NCI CTCAE v4.0 will be documented as well as vital signs and ECOG performance status. Analysis on samples collected will include hematology and clinical chemistry, tumor markers as well as plasma cytokines measured by BioplexPro™ Human Cytokine 27-plex Assay (Bio-Rad Laboratories Inc.). Assessment of tumor responses will be completed according to the response evaluation criteria in solid tumors (RECIST) 1.1/immune-based therapeutics RECIST based on computed tomography (CT) scans of the thorax, abdomen and pelvis as well as according to the positron emission tomography (PET) response criteria defined by the European Organization for Research and Treatment of Cancer (EORTC)/PET response criteria in solid tumors (PERCIST) (44, 45) using (18)F-fluorodeoxyglucose (FDG) PET scans (46). In addition to conventional measurements the FDG PET evaluation will include metabolic tumor volume (MTV) and total lesion glycolysis (TLG). Such imaging scans will be obtained prior to trial entry to be used as baseline (< 4 weeks of treatment start), at the 3 week mark, at completion of treatment (week 6) and again 6 weeks after treatment completion (week 12) and subsequently every 3^rd^ month until progression, or as per physician’s discretion. Finally, PROMs will be applied through questionnaires pre-, during and post-treatment. For assessment of T cell infiltration into tumor tissue some biopsy specimens will be sent to Veracyte in France (Luminy Biotech Enterprises) for immuno-oncologic diagnostics.

Samples will be collected prior to, during and after treatment. **Table 4** lists the samples and scans that are scheduled.

**Table 4:** Samples to be collected in the T-RAD study.

Tumor biopsies
Collected pre- and post-treatment. Post-biopsies will be obtained within one week of last T cell infusion and again and at 4-6 weeks post treatment completion and/or upon progression.
If sufficient tissue is available, three biopsies will be obtained at each timepoint and prioritized in the following order: 1. FFPE tissue 2. Snap-frozen tumor biopsies 3. Fresh tumor cells/tumor infiltrating lymphocytes prepared into a cell suspension
Blood samples will be collected pre-, during and post-treatment: 1. Peripheral blood mononuclear cells, processed with gradient centrifugation and frozen on liquid nitrogen (up to 10 samples scheduled) 2. Plasma/serum, separated and frozen (up to 17 samples scheduled) 3. Circulating tumor DNA/cells (only if sufficient resources available) 4. FDG PET/CT analysis at baseline (< 4 weeks of the first T cell infusion), again at the 3 week mark, at time of treatment completion (week 6), 6 weeks after treatment completed (week 12) and subsequently every 3^rd^ month or at progression

### Patient reported outcome measures

Participants will be asked to answer Quality of Life Questionnaires (QoL), at the start of the T cell infusions, at the 3 week mark, at the end of the treatment protocol at 6 weeks, at the 12 week follow-up and at the 24 week mark or as per physicians discretion. The questionnaires that will be applied are from the EORTC: Core Quality of Life Questionnaire, QoL-QLQ-C30 version 3.0 and Quality of Life Questionnaire-Lung Cancer 13, QoL-QLQ-LC13 version.

The QoL-QLQ-C30 is designed to assess the health-related quality of life of cancer patients. The questionnaire has 5 functional scales, nine symptom scales and a global health status, allowing for incorporation of physical, psychological, and social functioning into our assessments and refers to the patient’s perception of both the treatment and the illness.

The QoL-QLQ-LC13 is a lung-cancer specific questionnaire to be used in conjunction with the QLQ-C30 and is developed and validated in patients with lung cancer. This questionnaire includes domains of lung-cancer-related symptoms as well as treatment side effects. Both questionnaires will be scored according to the manual provided by EORTC (47).

### Withdrawal criteria

Patients may be withdrawn from the T-RAD study and its assessments at any time. Specific reasons for withdrawal include those seen in **Table 5**.

**Table 5:** Withdrawal criteria for the T-RAD study.

Withdrawal criteria
Voluntary withdrawal by the patient at any time
Safety reasons as determined by the responsible physician
Significant non-compliance to protocol as determined by the responsible physician.
Incorrect enrolment i.e., the patient does not meet the required inclusion/exclusion criteria
Patient is lost to follow-up
A female patient becoming pregnant
Disease progression
Deterioration of the patient’s condition that warrants discontinuation of the study treatment
Related Serious Adverse Event
Non-compliance to trial treatment

### Statistical analysis

The primary objective of the T-RAD study is safety. Statistical analysis will be performed after at least one patient has been enrolled, treated, and response has been evaluated. Per protocol, all end points will be analyzed in all treated patients. Safety analysis will include all patients who received treatment. Safety monitoring is the main aim of this trial and thus a power analysis is not applicable beforehand, but the number of patients is similar, to that analyzed in comparable trials (31). The secondary objectives focus on clinical response and are based on PFS, ORR, TTP and OS. For this the Kaplan-Meier methodology will be used. Given the exploratory nature of this phase I study, no adjustments for multiple comparisons will be made.

The samples size is based on clinical considerations and the dose-escalation design. Descriptive statistics include means with standard deviations or medians with minimum and maximum for continuous variables. Counts and percentages will be provided for categorical variables. Missing data will not be imputed unless specified. Confidence intervals will be used where appropriate. All statistics will be done as advised by statisticians.

### Ethics

The treatment protocol, including the patient information and informed consent forms to be used, must be approved by the regional ethics committee before treatment is initiated. Treatment under this clinical study protocol will be conducted in accordance with ethical principles that have their origin in the Declaration of Helsinki. The responsible physician will inform the ethics committee of any major amendments to the treatment protocol as per national requirements.

## Discussion

The treatment of cancer is currently undergoing very exciting revolutionary changes. Immunotherapy has taken the stage by storm and the number of new treatments being researched has exploded. However, ACT to date has mainly been dominated by the successful treatment of refractory hematological malignancies using CARs, and no TCR has to date been approved for such use. Noteworthy in this context though is the newly FDA approved bispecific T-cell engager, tebentafusp, which is used in unresectable/metastatic uveal melanoma and targets the melanoma-associated antigen (gp100) (48). Treatment with CARs has high rates of CRS and more serious, and even fatal, variants of immune cell overstimulation and cytokine storm can occur, such as immune effector cell-associated neurotoxicity syndrome (ICANS). Additionally, off-target effects and/or on-target, off-tumor effects of CARs have caused severe and even fatal reactions in some reports (13, 14). Unfortunately, cellular immunotherapy has not yet made significant progress in the realm of solid tumors, although some encouraging reports have been published (4). TCR-based therapies could be more efficient than CARs against solid tumor, and the number of clinical trials testing different TCRs is increasing (49, 50). Compared to CAR therapy, TCR therapy greatly expands the number of targetable tumor antigens by including all proteins presented on the cell surface in the context of MHC, by requiring less abundancy of the target antigen and by reducing the risk of cross-reactivity with healthy cells (51). Our unique platform of using unmodified/unenhanced TCRs that originate from survivors from cancer vaccine trials greatly decreases the risk of off-target toxicity given prior thymic selection of the TCRs. This is, to our knowledge, unprecedented in the field of TCR therapies, providing a safer option where serious side effects could be lethal. Furthermore, prior TCR therapies have often been limited by a very narrow applicability due to MHC restriction.

The Radium-4 recognizes hTERT that is an attractive target for TCR immunotherapy given how commonly it is overexpressed in solid cancers, including metastatic NSCLC (9, 52–55) where survival is poor. Importantly, the Radium-4 is restricted by an MHC class II allele (HLA-DP04) that is common in the Caucasian population making the Radium-4 TCR potentially applicable to a vast number of solid cancer patients, unlike most prior TCR immunotherapy studies.

**Figure 7:**
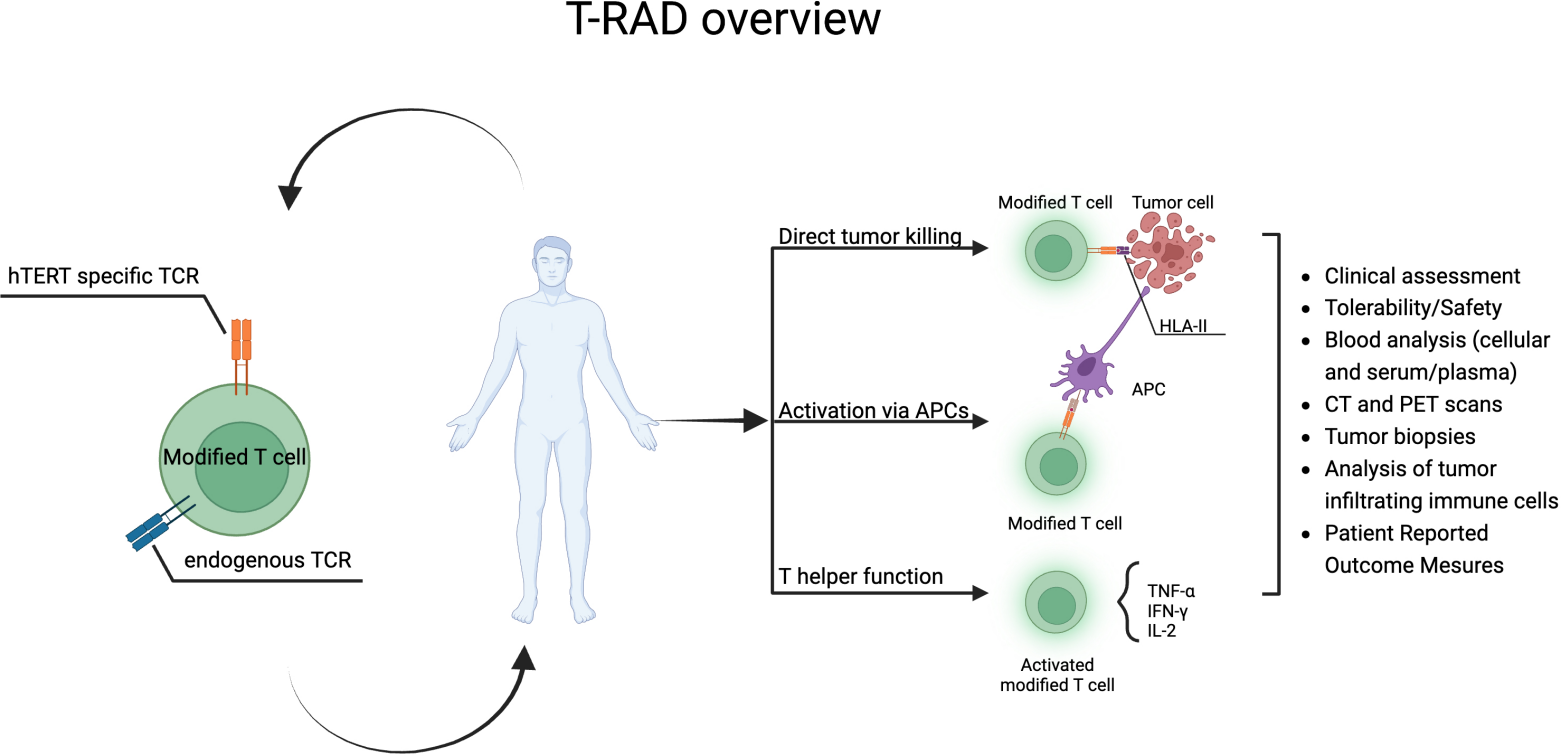
T-RAD clinical study overview. Created using BioRender.com.

Based on the available data the risk benefit profile for Radium-4 T cell immunotherapy is excellent and no toxicity to hematopoietic stem cells was observed in our preclinical study (20), however, an effect on germ cells cannot be ruled out. One prior publication on an hTERT TCR that was MHC class I restricted demonstrated an effect on the granulocytic compartment (56, 57) but was otherwise well tolerated and effective in preclinical studies. Only one published study using TCR immunotherapy has applied an MHC class II restricted TCR with a good safety profile and some efficacy (58). The loss of MHC class I expression on cancer cells presents a challenge to cancer immunotherapy. MHC class II restricted T cells can indirectly recognize TAA on antigen-presenting cells (APCs) and thus provide help to cytotoxic T cells and broaden the immune response by epitope spreading. This can alter the tumor microenvironment and create an avalanche of immune responses leading to the destruction of the cancer cells. Intrinsic cancer-cell MHC class II expression has been shown to regulate both the tumor immune microenvironment and sensitivity to anti-PD-1 treatment in lung cancer (59). This multifaceted role of CD4 T cells is important for a sustained anti-tumor response (60, 61) as recently reviewed by Oh et al (62). Finally, MHC class II restricted T cells can also directly recognize their cognate antigen on cancer cells (63–65). Studies, including ours, have demonstrated the direct cytotoxic effects of CD4 T cells through TNFα and IFNγ and granzyme B production from cytotoxic hTERT specific CD4 T cells (16). Furthermore, a recent publication showed similar cytotoxicity for CD4 tumor-infiltrating-lymphocytes (TILs) in bladder cancer (61). Studies also suggested that tissue resident CD4 T cells are important targets for immune checkpoint inhibitor therapy such as in lung cancer (66, 67) See **Figure 7** for summary of the T-RAD study.

By application of mRNA electroporation, we ensure transient expression of the Radium-4 TCR on the infused autologous T cells. Thus, there is no permanent genetic alteration of the patient T cells. This also allows for quick termination of treatment and side effects by discontinuation of T cell infusions. This is a key safety element to the T-RAD trial since it is a first-in-human study and both CRS and cross-reactivity can never be completely ruled out prior to a clinical study despite our best efforts and negative results to date in our prior study (20) (ClinicalTrials.gov:NCT03431311, manuscript in preparation). This is different from the vast majority of prior ACT trials where permanent viral transduction has been applied.

Monitoring of plasma cytokines will give insight into immune cell activation following autologous T cell infusions. With the ability to collect metabolic information, FDG PET has become an increasingly important tool for accurate detection of malignancies and early response evaluation in patients with cancer, and thus will provide valuable information in our study. Immune cell characterization in tumor biopsies will be of essence in this study since it allows for evaluation of changes in the tumor microenvironment and the potential applicability of this treatment modality.

In summary we present a clinical study using an MHC class II restricted hTERT specific TCR, Radium-4, to treat metastatic NSCLC in patients who have no other treatment options. This is a first-in-human study and tolerability is the primary endpoint in the study. The study presents a few novelties in this realm of immunotherapy, from that of using a TCR restricted by a commonly expressed MCH class II allele, to the origin of the TCR from a survivor of a vaccine study and transient expression through mRNA electroporation. All being critical in minimizing the risk of toxicity in this trial. The primary objective of this study is to assess tolerability and safety of the T-RAD treatment and hopefully demonstrate a treatment option for solid tumors by opening the gates for TCR immunotherapy.

## Data availability statement

Publicly available datasets were analyzed in this study. This data can be found here: The Genotype-Tissue Expression (GTEx) project and the TCGA research network.

## Ethics statement

The studies involving human participants were reviewed and approved by South-Eastern Regional Ethical Committee (approval references: 2019/121, 2016/2247, and 2013/624 for human material). The patients/participants provided their written informed consent to participate in this study.

## Author contributions

SM and EI wrote the draft of the manuscript. SM, EI, and PW designed the figures. All authors are involved in the design, planning, or preparation of the clinical trial. All authors have read and approved the final manuscript.

## Funding

This research was funded the South-Eastern Norway Regional Health Authority (Grant number 2017075), the Research Council of Norway (Grant number: 244388), and the Radium Hospital Research Foundation.

## Acknowledgments

We thank our team of engineers: Marianne Lundby, Kirsti Hønnåshagen, Lisbeth J. Skoge, Guri Solum for excellent work on T cell expansions and Dr. Marit Renée Myhre, Birthe Mikkelsen Saberniak and Dr. Jens Andreas Lindin Jørgensen for GMP-grade mRNA preparation. We thank Hedvig Vidarsdotter Juul for the functional testing of electroporated T cells. We are grateful to pharmacist Cecilie Nguyen for assistance with documentation.

## Conflict of interest

The authors declare the following potential conflict of interest: GK, SW, and EI are named inventors on a patent on Radium-4 TCR WO2019166463.

The remaining authors declare that the research was conducted in the absence of any commercial or financial relationships that could be construed as a potential conflict of interest.

## Publisher’s note

All claims expressed in this article are solely those of the authors and do not necessarily represent those of their affiliated organizations, or those of the publisher, the editors and the reviewers. Any product that may be evaluated in this article, or claim that may be made by its manufacturer, is not guaranteed or endorsed by the publisher.
